# Regional distribution of cytochrome c oxidase activity and copper in sclerotic hippocampi of epilepsy patients

**DOI:** 10.1002/brb3.1986

**Published:** 2020-12-07

**Authors:** Miloš Opačić, Aleksandar J. Ristić, Dragoslav Sokić, Vladimir Baščarević, Savo Raičević, Slobodan Savić, Maja Zorović, Marko Živin, Vid Simon Šelih, Ivan Spasojević, Danijela Savić

**Affiliations:** ^1^ Department of Life Sciences Institute for Multidisciplinary Research University of Belgrade Belgrade Serbia; ^2^ Centre for Epilepsy and Sleep Disorders Neurology Clinic Clinical Centre of Serbia Belgrade Serbia; ^3^ Institute for Neurosurgery Clinical Centre of Serbia Belgrade Serbia; ^4^ Institute of Forensic Medicine ‘Milovan Milovanović’ Medical School University of Belgrade Belgrade Serbia; ^5^ Brain Research Laboratory Institute of Pathophysiology Medical Faculty University of Ljubljana Ljubljana Slovenia; ^6^ Department of Analytical Chemistry National Institute of Chemistry Ljubljana Slovenia; ^7^ Department of Neurobiology Institute for Biological Research ‘Siniša Stanković’ – National Institute of Republic of Serbia University of Belgrade Belgrade Serbia

**Keywords:** copper, cytochrome c oxidase, hippocampal sclerosis, LA‐ICP‐MS, temporal lobe epilepsy

## Abstract

**Introduction:**

Disruption of copper homeostasis and dysfunction of mitochondria have been documented in sclerotic hippocampi (HS) of patients with mesial temporal lobe epilepsy (mTLE). However, a potential link between these pathological changes has not been tackled so far. Herein, we analyzed regional distribution of neuron somata density, copper concentration, and the activity of cytochrome c oxidase (CCO), a component of mitochondrial electron transport chain and copper‐containing metalloprotein, in HS.

**Methods:**

Histochemical staining and laser ablation inductively coupled plasma mass spectrometry were carried out to construct comparable maps of these parameters in coronal sections of hippocampi of 3 mTLE‐HS patients and 3 control subjects.

**Results:**

Copper levels were decreased in all regions of HS with pyramidal neuron somata. CCO activity was significantly reduced in stratum pyramidale (PY) 1 and cornu Ammonis field 4, the two regions with significant reduction in neuron somata density. CCO activity was also lower in layers that contain apical dendrites of pyramidal neurons and mossy fibers. It appears that copper deficiency in PY2 and PY3 comes before CCO activity reduction and neuronal loss. A strong positive correlation was found between neuron density, Cu concentration, and CCO activity.

**Conclusions:**

Presented results imply that pathological alterations in Cu and energy metabolism could be involved in the development of HS. A limitation of this study was the relatively small number of patients. However, presented results underline copper deficiency as a component of pathological mechanisms of epilepsy and warrant further investigation of cuproproteins and members of copper transport machinery.

## Graphical abstract

Regional distribution of neuron somata density, copper concentration, and the activity of cytochrome c oxidase in coronal sections of sclerotic hippocampi of epilepsy patients and controls.

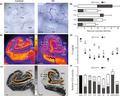

## 1 INTRODUCTION

The loss of homeostasis of specific metals is implicated in pathophysiology of mesial temporal lobe epilepsy associated with hippocampal sclerosis (mTLE‐HS), the single most frequent human focal epilepsy. In addition to widely recognized but still controversial role of mossy fibers zinc in hyperexcitability and circuit rearrangements in HS (Coulter, 2000; Frederickson et al., 2005), emerging data imply that copper may be involved in epileptogenesis as well. The total Cu concentration in hippocampal tissue of mTLE‐HS patients is significantly lower than in age‐matching controls (Ristić et al., 2014). Further, laser ablation inductively coupled plasma mass spectrometry (LA‐ICP‐MS) imaging of sclerotic hippocampi has shown that Cu concentration is positively correlated with neuron density in stratum pyramidale of cornu Ammonis (CA) region 1 (PY1), with areas of total neuron loss showing significantly reduced Cu concentrations (Opačić et al., 2017). It is important to point out here that low Cu concentrations in the brain have been linked to seizures in Menkes disease and in hypocupremia that is caused by dietary restriction or chelating drugs (Benbir et al., 2010; Lozano Herrero et al., 2012; Schlief et al., 2006). The latter implies that decrease of Cu concentration is not just an epiphenomenon of epileptic phenotype. It is worth mentioning that Cu and Zn may have intertwined roles in HS. For example, metal‐regulatory transcription factor 1, which is strongly implicated in HS pathogenesis and represents the key controller of cellular Cu homeostasis, is activated by Zn (Selvaraj et al., 2005; van Loo et al., 2015).

To understand the involvement of Cu in mTLE‐HS, it is important to identify Cu metalloproteins that show altered function in sclerotic hippocampi. So far, we have found that there are no changes in the activity and level of copper‐zinc superoxide dismutase, a central cytosolic antioxidative enzyme, in HS. On the other hand, the activities and/or levels of manganese superoxide dismutase and other components of mitochondrial antioxidative system were significantly higher than in controls (Ristić et al., 2015). In line with this, a recent study has shown that hippocampal mitochondria are exposed to oxidative stress in mTLE‐HS (Volmering et al., 2016). A number of other findings also link mTLE‐HS pathology with mitochondrial dysfunction, including the following: (a) different metabolic abnormalities in the hippocampus, such as decreased activity of complex I of electron transport chain (ETC) in the areas with ongoing sclerotic processes (Kunz et al., 2000), decreased concentrations of mitochondrial metabolites in epileptogenic foci (Mueller et al., 2003; Vielhaber et al., 2008), interictal glucose hypometabolism (O'Brien et al., 1997), and abnormal NAD(P)H transients in response to ex vivo stimulation (Kann et al., 2005); (b) presence of mitophagy in sclerotic hippocampi (Wu et al., 2018); and (c) variations in mitochondrial DNA that are more frequent in mTLE‐HS, and that may affect conformation of several proteins in complex I and ATP synthase (Azakli et al., 2013; Gurses et al., 2014). Cytochrome c oxidase (CCO; ETC complex IV) represents a crossroad between mitochondria and Cu metabolism. CCO contains three Cu ions in two centers (binuclear Cu_A_ and mononuclear Cu_B_), which are essential for passing electrons from complex III and cytochrome c to O_2_ (Yoshikawa et al., 2011). CCO deficiency and inhibition have been related to the development of seizures and epileptic phenotype (Hallmann et al., 2016; Miles et al., 2015; Rezek & Moore, 1986; Yamamoto & Tang, 1996; Zsurka & Kunz, 2015). Finally, a decrease in CCO activity has been observed in seizure onset zones in focal cortical dysplasia (Miles et al., 2015).

The aim of this study was to compare regional distributions of neuron somata density, Cu concentration, and CCO activity in hippocampi of mTLE‐HS patients and controls, and to establish a potential relation between these parameters in HS. Detailed maps of Cu and neuron somata density and CCO activity were built using LA‐ICP‐MS imaging and histochemical methods.

## 2 MATERIAL AND METHODS

### 2.1 Patients and sample collection

This study was performed on three sclerotic hippocampi that were removed *en bloc* in the course of anterior temporal lobectomy with amygdalohippocampectomy on pharmacoresistant (≥3 antiepileptic drug failures) mTLE‐HS patients, at the Clinic of Neurosurgery, Clinical Center of Serbia; and on three control hippocampi that were obtained <10 hr *post mortem* from individuals without a history of neurological diseases and brain injury, at the Institute of Forensic Medicine, Medical School, University of Belgrade. Data on all subjects are provided in Table S1. HS was confirmed by MRI findings and histopathology analyses, and classified as type 1 according ILAE criteria (Blümcke et al., 2013). Iatrogenic metal intake was negligible during the two‐year period prior to the surgery. The research was performed in accordance with the 1964 Declaration of Helsinki of the World Medical Association and its later amendments and has been approved by the Ethics Committee on Human Research at the Clinical Center of Serbia. Written informed consent was obtained from each patient. Samples (posterior part of the head of the hippocampus, thickness <1 cm) were snap frozen in liquid N_2_ at extraction and stored at −80°C.

### 2.2 Tissue preparation

A series of coronal sections (16 µm thick) of each hippocampus sample were cut on cryostat and collected onto adhesive microscope slides (SuperFrost Ultra Plus, Thermo Scientific) for cresyl violet (CV – Nissl) staining and CCO histochemistry, or on uncoated glass slides for LA‐ICP‐MS. Sections were collected in the series of three with one for each method. All sections were left to dry in air and stored at −20°C until further analyses. Sections were protected with metal‐free Ultralene foils.

### 2.3 Cresyl violet staining and CCO histochemistry assay

Cresyl Violet staining was performed to label neuronal cell bodies and to identify and mark regions in hippocampal anatomy. Post‐sectioning fixation was performed in 4% paraformaldehyde for 10 min at 4°C. Sections were incubated in 0.1% CV acetate stain, distained in 70% ethanol, dehydrated through 96% and absolute ethanol, cleared in xylene and mounted in DPX. CV‐stained sections were examined by Zeiss Axiovert microscope (Carl Zeiss). CCO histochemistry activity assay was performed as described previously (Wong‐Riley, 1979). The protocol uses 3,3’‐diaminobenzidine as electron donor to cytochrome c, which is than oxidized by CCO to generate visually detectable reaction product. Sections were fixated in 4% paraformaldehyde for 20 min at 4°C and incubated in 50 mM phosphate buffer solution (pH 7.4) that was supplemented with 3,3’‐diaminobenzidine (0.02%), sucrose (2%), cytochrome c type III (0.03%), catalase (0.015%), (NH_4_)_2_Ni(SO_4_)_2_ (0.03%), and CoCl_2_ (0.03%), at 44°C with gentle shaking for 70 min. The reaction was stopped by rinsing the sections in cold 100 mM phosphate buffer solution (3 × 5 min). All chemicals were purchased from Sigma‐Aldrich. Slides were dehydrated, cleared, and mounted following the same protocol as for CV staining. Microscopic slides with CCO activity‐stained sections were trans‐illuminated with the light‐box (Northern Light Technologies), for semi‐quantitative densitometry. Images of each sample were acquired under identical conditions by sensitive black and white DAGE72E video camera (DAGE‐MTI), and MCID image acquisition software (MCID Analysis, http://www.mcid.co.uk, RRID:SCR_014278).

### 2.4 LA‐ICP‐MS instrumentation

LA‐ICP‐MS instrumental setup was comprised of LA system (193 nm ArF* excimer laser; Analyte G2 Teledyne Photon Machines Inc.,) that was equipped with active two‐volume ablation cell (HelEx II), and Aerosol Rapid Introduction System (ARIS, Teledyne CETAC Technologies) to achieve fast LA aerosol washout times (typically ca. 20 ms, FW0.01 M). The LA unit was coupled with quadrupole ICP‐MS instrument (Agilent 7900x, Agilent Technologies). Ar makeup gas was added to ARIS torch adaptor before ICP torch (0.8 L/min). Instrumental settings for LA and ICP‐MS imaging were based on assumption of optimized image quality, fast ablation and prevention of imaging artifacts such as image blur, smear, aliasing, and noise (van Elteren et al., 2019). We used the following instrumental settings: LA – beam size: 20 µm square shape; repetition rate: 294 Hz; scanning speed: 588 µm/s; fluence: 0.36 J/cm^2^; He flows: 0.3 L/min (cup), 0.3 L/min (cell). Ablation mode‐continuous line scanning (20 µm spacing) with dosage of 10 shots/pixel. ICP‐MS – mode: transient resolved analysis; RF power: 1,500 W; Ar makeup flow to ARIS adaptor: 0.8 L/min; isotope reported: ^63^Cu; acquisition time: 34 ms. These settings provide elemental images with square pixels (20 × 20 µm), at imaging rate of 106 kilo pixels/h. Data processing to obtain raw elemental images was performed using the software package HDIP (v1.3.1.r0; Teledyne CETAC Technologies). For calibration of raw elemental images, calibration standards were prepared from gelatin using a procedure reported previously (Šala et al., 2017). In short, gelatin solution (10%) was spiked by the Cu solution to known final concentrations. Droplets were deposited on glass slides and dried using optimized procedure to prevent migration of ions in the droplet during drying/setting of the gelatin gel due to Marangoni and coffee‐stain effects, thus providing for ultimately homogeneous distribution of spiked elements in the dried gelatin. Areas on spiked gelatin were subsequently ablated to obtain element calibration responses while using the same LA‐ICP‐MS instrumental settings as for the samples under investigation. Calibration response factors were obtained and used to convert raw elemental images into calibrated ones using ImageJ software (https://imagej.nih.gov/ij, RRID:SCR_003070). Further, the applied fluence settings generated ablation of 2 µm of sample depth. In this way, sample material was not completely removed from the glass slides, so the equal thickness of different samples and calibration gelatin droplets was not needed. In addition, ablation with low fluence prevents unwanted physical effects on the ablated material, such as: fraying of edges, random ejection of material from proximal locations, delamination, ejection, and redeposition of larger particles. In addition, the approach provides superior S/N ratios under high imaging rate that was applied here .

### 2.5 Image analysis

Images were analyzed in ImageJ software. Regions of interest (ROI) in coronal sections were outlined according to Duvernoy (2005) by an experienced neuropathologist: stratum pyramidale of CA fields 1, 2, and 3 (PY1, PY2, PY3), CA field 4 (CA4), strata radiatum and lacunosum of CA (RL), stratum moleculare of CA (M), stratum moleculare of gyrus dentatus (GDM), granular cell layer (GRCL), and subiculum (SUB). For the analysis of sub‐regional distribution and correlation of neuron somata density (number of cells per mm^2^), Cu concentration, and CCO activity, additional ROI were outlined as polygonal, equi‐length (500 µm) regions alongside stratum pyramidale anatomical edge in PY3, PY2, PY1, and SUB. Three images (CV, copper level, and CCO activity) from consecutive sections of each hippocampus were overlaid and aligned according to neuron somata areas, activity regions, and section edges as guiding points. ROI were marked in CV‐stained images by a freehand selection tool. Delineation was transferred to copper and CCO images. It is important to note that the original images of CCO activity were changed in ImageJ to 8‐bit grayscale type and inverted with the 0 (white) to 255 (black) scale. Relative optical density (ROD) was measured in each ROIas mean gray value. Background ROD of the white matter in the same section was subtracted. All values were normalized to ROD of SUB.

### 2.6 Statistical analysis

Statistical analysis was performed in IBM SPSS 25 software (SPSS, http://www‐01.ibm.com/software/uk/analytics/spss, RRID:SCR_002865) and GraphPad Prism 5 software (GraphPad Prism, http://www.graphpad.com, RRID:SCR_002798). Homogeneity of variance was tested using Levene's test. *p*‐values were determined using independent samples *t* test, two‐tailed (control versus HS comparison), and one‐way ANOVA followed by Dunnett's post hoc test (SUB versus other ROI within HS or control group). Data are presented as mean ± standard error (SE). Correlation analysis was performed using Spearman's rank correlation coefficients (ρ). Data were considered statistically significant for *p* < .05.

## 3 RESULTS AND DISCUSSION

**FIGURE 1:**
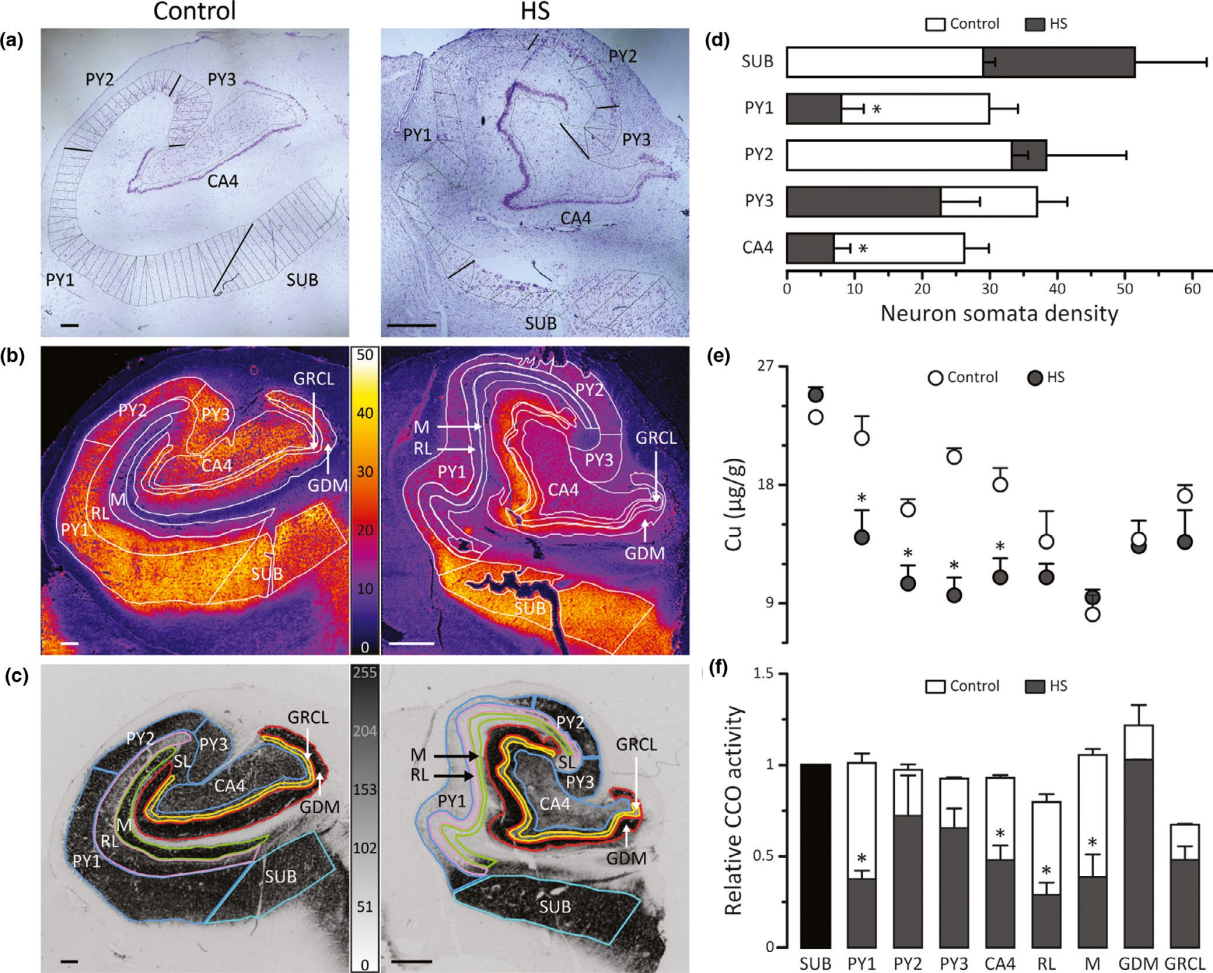
Micrographs and quantitative analysis of regional distribution of neuron somata density, Cu concentration and CCO activity in the coronal sections of control and sclerotic hippocampi (HS). (a) CV staining of neurons, with marked SUB, PY1, PY2, PY3, and CA4 fields, and equi‐length areas that were further used in correlation analysis. (b) LA‐ICP‐MS pseudo‐color maps of distribution of Cu concentrations. Pseudo‐color scale: 0–50 µg of Cu per g of tissue. (c) CCO activity distribution with outlined ROI that correspond to regions of hippocampal anatomy. Gray scale (0–255) of ROD indicates CCO activity. (d) Neuron somata density (number of somata per mm^2^) in specific ROI. (e) Distribution of Cu concentrations in different ROI; (f) CCO activity in different ROI normalized to CCO activity in SUB (black bar) in the same sample. *p < .05, statistical significance compared to the same region in controls, as determined by independent samples *t* test. Data are expressed as mean ± SE. Scale bar: 1 mm. SUB, subiculum; PY1, PY2, PY3 ‐ stratum pyramidale of cornu Ammonis (CA) fields 1, 2 and 3; CA4, CA field 4; RL, strata radiatum and lacunosum of CA; SL, stratum lucidum; M, stratum moleculare of CA; GDM, stratum moleculare of gyrus dentatus; GRCL, granular cell layer

**FIGURE 2:**
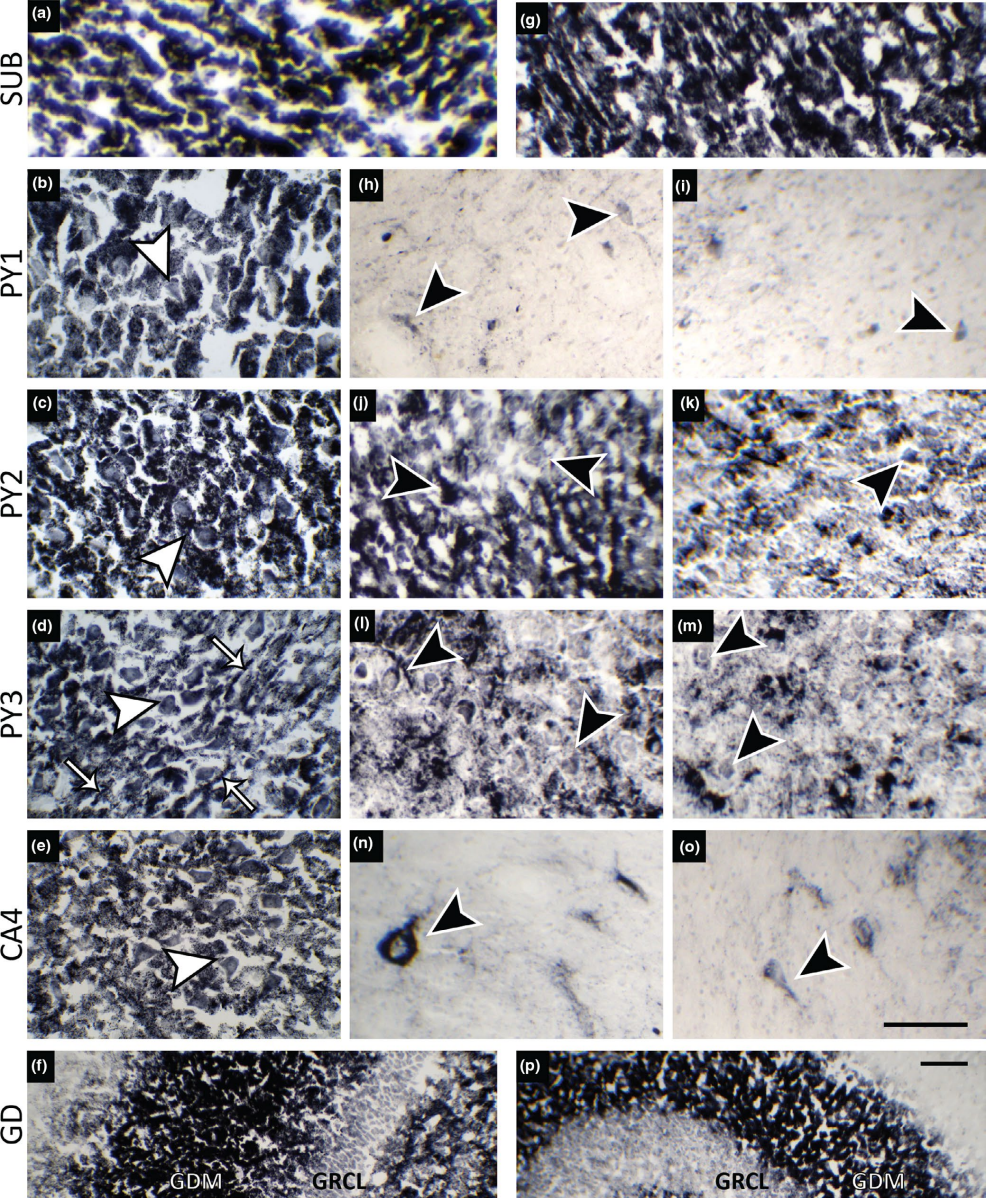
Micrographs of CCO histochemical reaction product (CCO activity) in control hippocampi and hippocampi from mTLE‐HS patients. (a–e) CCO activity is located in neuron somata of SUB, PY1‐3 and CA4 (white arrowheads), and in processes around PY3 neurons (white arrows) of control sections. (g–o) SUB and surviving neurons in PY1‐PY3 and hypertrophied neurons in CA4 (black arrowheads) in HS. GDM in control hippocampi (f) and HS (p), with no obvious difference in metabolic activity. Scale bar (100 µm) in panel (o) applies to panels A‐E and G‐N. Scale bar (100 µm) in panel (p) applies to panel F. SUB, subiculum; PY1, PY2, PY3 ‐ stratum pyramidale of cornu Ammonis (CA) fields 1, 2, and 3; CA4, CA field 4; GDM, stratum moleculare of gyrus dentatus; GRCL, granular cell layer

Figure 1 and Figure S1 show CV micrographs and maps of Cu concentration and CCO activity in control and HS hippocampi. It can be observed that the principal neuronal regions were severely affected in HS. Neuron somata density in PY1 and CA4 was ~ 70% lower than in controls. PY3 showed a similar trend, whereas PY2 did not show significant neuronal loss (Figure 1d). Pertinent to this, PY2 is known to be less susceptible to sclerosis than other PY regions (Houser, 1992; Thom et al., 2005). It is important to point out that SUB represents a region that is analogous to PY and that maintains a well‐preserved neuron population in HS (Opačić et al., 2017; Thom et al., 2005). Further, there was no difference in neuron somata density between PY1‐PY3 and CA4 compared to SUB in controls, whereas in HS, neuron density was lower in PY1 and CA4 than in SUB (Table S2). In addition, it appears that neuron density in SUB is higher in HS than controls (Figure 1d), which has been reported previously (Alonso‐Nanclares et al., 2011), and may be related to a functional compensation for neuron loss in sclerotic areas. Spatial Cu distribution is presented in Figure 1b. Cu concentration was higher in areas that contain neuronal somata than neuropil. The highest concentration of Cu in both control and HS group was measured in SUB, whereas the lowest was in M, with no significant differences between controls and HS (Figure 1e). All layers with pyramidal neurons showed significantly lower Cu in HS than controls. It is important to stress out that PY2 showed a significant drop in copper concentration whereas no decrease in neuron density was present. The layers of GD lamina (GDM and GRCL) showed similar Cu concentrations in both groups (Figure 1e). In HS, all regions/layers had significantly less Cu than SUB (Table S2). Distribution of CCO activity is presented in Figures 1c and 2. CCO activity in SUB was similar for HS and controls. SUB was used as internal reference for normalization of CCO activity in other regions (Figure 1f). HS was related to significant reduction of CCO activity in PY1, CA4, RL, and M. In controls, all regions of hippocampal anatomy had similar CCO activity. On the other hand, a widespread reduction in CCO activity was noticed in HS (Table S2). It is important to note that CCO histochemical reaction product (i.e., CCO activity) was mainly localized in neuron somata (Figure 2a–e, control; Figure 2g–o, HS), as well as in GDM, a layer that predominantly contains neuronal processes (Figure 2f,p). In areas of total neuronal loss in PY1, sporadic and weak CCO activity was registered in surviving pyramidal neurons (Figure 2h,i). CA4 is another region that is heavily affected by neuronal loss in HS type 1 (Blümcke et al., 2013). CCO activity staining revealed that this region is characterized by the presence of neurons that show several times enlarged bodies compared to control (Figure 2n,o). This phenomenon has been observed previously in TLE‐HS (Ryufuku et al., 2011). Visualization of CCO activity also showed that neuron somata in PY2 in control hippocampi are densely packed (Figure 2c), whereas in HS, this layout was partially lost (Figure 2j,k). Furthermore, stratum lucidum and processes that surround soma of surviving PY3 neurons in HS showed a significant drop (~50%) in CCO activity (Figure 2d,l,m).

**FIGURE 3:**
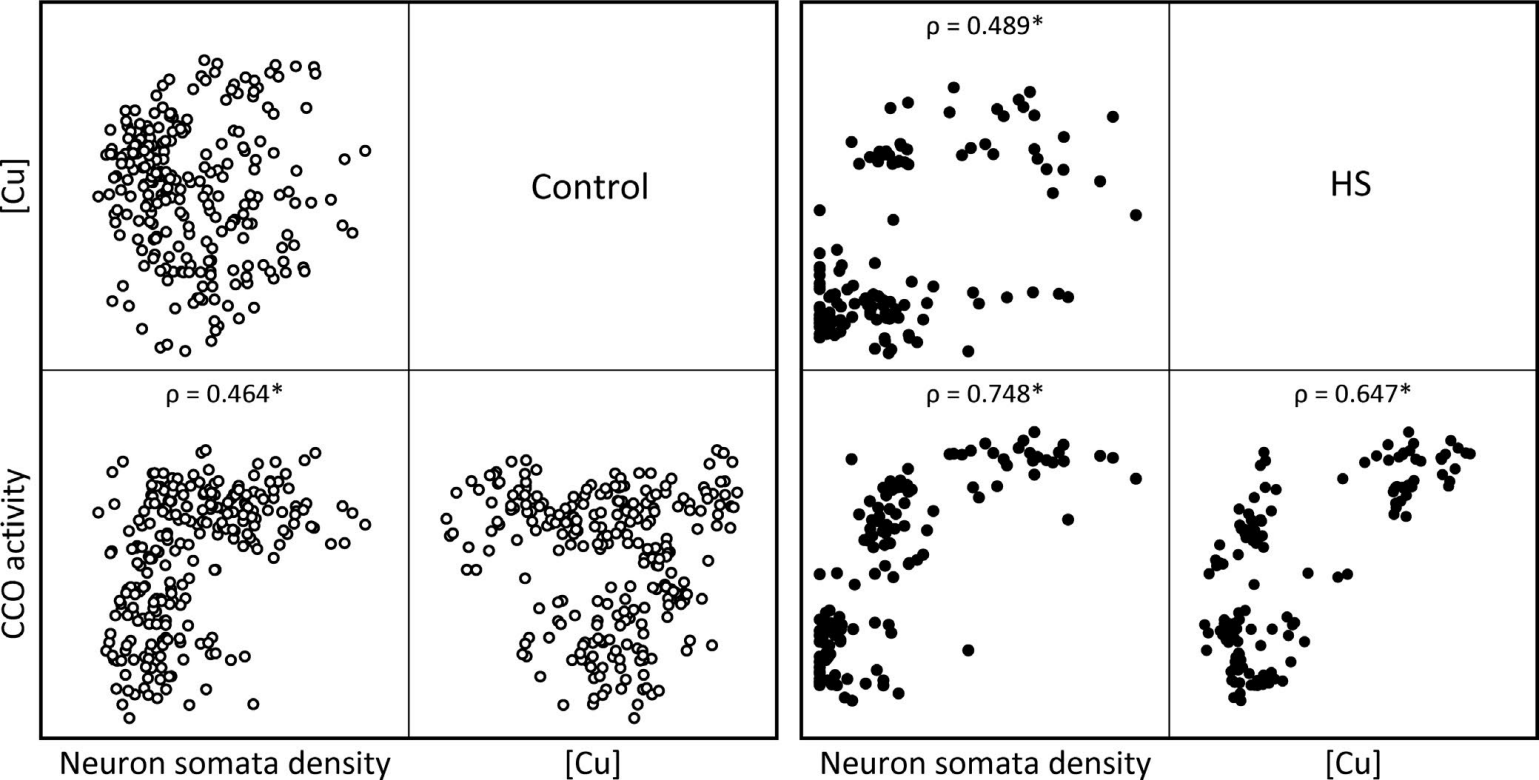
Correlations between neuron somata density, Cu concentration [Cu], and CCO activity in pyramidal cell regions (SUB + PY1‐PY3), in control and HS. Scatter plots of Spearman correlation analysis are presented in quadrants. ρ, Spearman's rank correlation coefficient. *p < .05, significant correlation.

Next, we analyzed the distribution and correlation of neuron somata density, Cu concentration and CCO activity along pyramidal cell regions SUB and PY1‐PY3 (Figure 3 and Figure S2). A significant correlation between neuron somata density and CCO activity was found in controls and HS. In HS, a strong positive correlation was determined also between Cu concentration and CCO activity, and Cu concentration and neuron somata density (Figure 3). Finally, a positive correlation between CCO activity and Cu concentration was determined in HS in each pyramidal cell region taken separately (Figure S3).

Our results strongly imply that HS, CCO, and Cu are related, and substantiate previous findings that have related seizures and epileptogenesis with dysfunctional mitochondria and CCO deficiency and the loss of Cu homeostasis (Hallmann et al., 2016; Miles et al., 2015; Rezek & Moore, 1986; Yamamoto & Tang, 1996; Zsurka & Kunz, 2015). CCO showed decreased activity in regions with pyramidal neurons somata that were most affected by sclerosis—PY1 and CA4. In relation to this, a significantly lower CCO activity was observed in RL and M, regions that contain proximal and distal parts of apical dendrites of CA pyramidal neurons. It is important to note that pyramidal neurons represent energetically most active cells in the hippocampus (Kann et al., 2014). In contrast, GDM, which is occupied mostly by axons from entorhinal cortex and dendrites from dentate granule cells, appears to be unaffected. Further, HS was related to significant decrease in Cu concentration in all regions with pyramidal neurons. This is in accord with our previous findings on the relation between HS and Cu concentration decrease (Opačić et al., 2017; Ristić et al., 2014). PY2 and PY3 did not show a change in neuron density and CCO activity, whereas a significant drop in copper concentration was observed. This implies that the decrease in Cu concentration might precede and induce the disturbance of energy homeostasis and/or loss of neurons. It is important to point out that a limitation of this study was the relatively small number of patients which may generate bias. Other potential sources of bias may include the use of *post mortem* tissue, limited comparability of LA‐ICP‐MS results in a given study with other publications, and effects of diet. It is tempting to speculate that alternations in some aspects of Cu metabolism, such as transport to mitochondria and/or CCO, may affect CCO levels and energy status in hippocampus that may further lead to HS. Further research of Cu transport machinery and metalloproteins on a larger number of patients with HS is warranted.

## CONFLICT OF INTEREST

The authors declare that they had no financial or commercial conflicts of interest.

## AUTHOR CONTRIBUTION

IS, MO, DSa, and AJR have made substantial contributions to conception and design, acquisition, analysis, and interpretation of data. AJR, DSo, VB, SR, and SS participated in the clinical practice, including diagnosis, treatment, consultation, and sample acquisition. MZ, MŽ, and VSŠ have made substantial contributions to acquisition and analysis of data. IS, MO, and DSa have been involved in drafting the manuscript. All authors critically reviewed and approved the final version of the manuscript.

### PEER REVIEW

The peer review history for this article is available at https://publons.com/publon/10.1002/brb3.1986.

## Supporting information

Supplementary Material

## Data Availability

The data that support the findings of this study are available in the supplementary material of this article.
