# Supplementary material for: Regional distribution of cytochrome c oxidase activity and copper in sclerotic hippocampi of epilepsy patients
Source: Brain Behav. 2020 Dec 7;11(2):e01986. doi: 10.1002/brb3.1986 (PMC7882178; doi:10.1002/brb3.1986)
Supplement: Supplementary file 1 — Supplementary Material [file BRB3-11-e01986-s001.pdf]

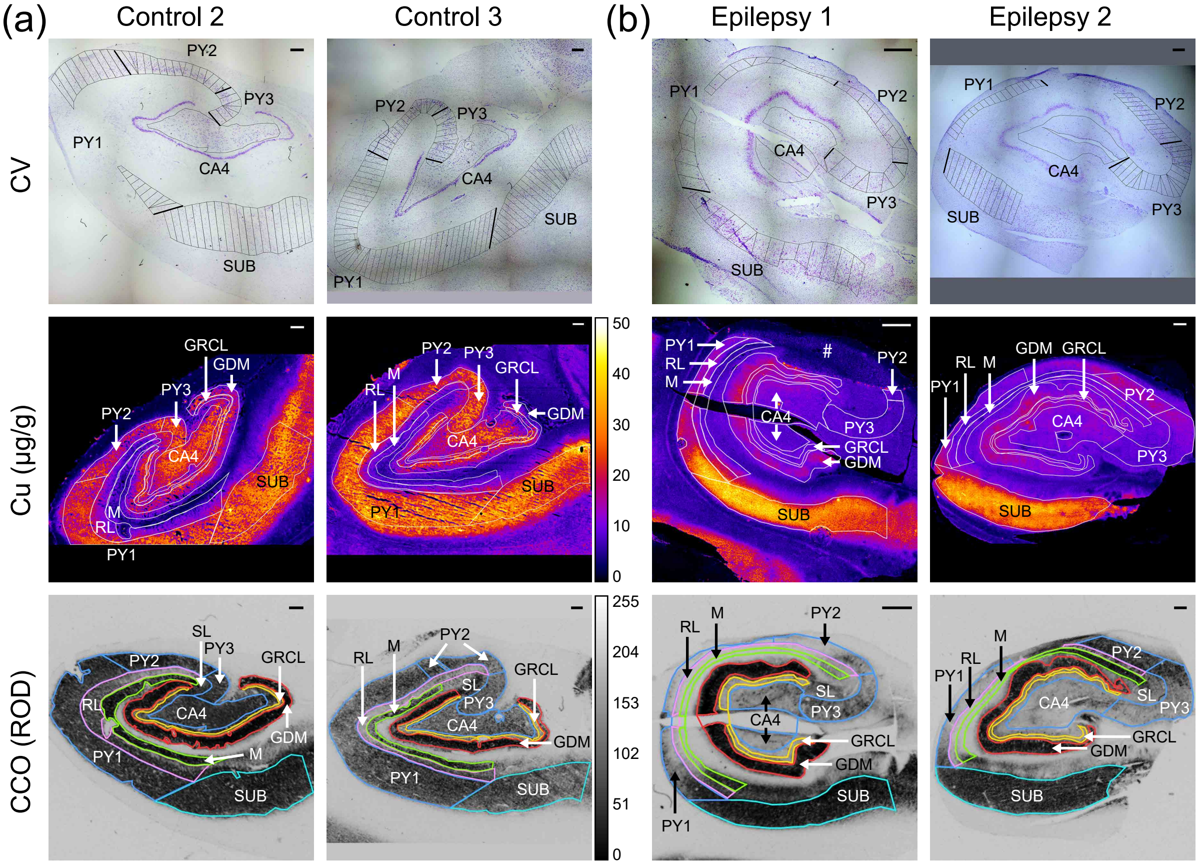

(a)
Control 2
Control 3
(b)
Epilepsy 1
Epilepsy 2
CV
Cu (μg/g)
CCO (ROD)
PY1
PY2
PY3
CA4
SUB
GRCL
GDM
RL
M
SL
#
50
40
30
20
10
0
255
204
153
102
51
0

FIGURE S1 Micrographs showing regional distribution of neuron somata density (CV), Cu concentration and CCO activity in the coronal sections of 2 control (a) and 2 hippocampi from mTLE-HS patients (b). Micrograph of the CV-stained section show delineated SUB and principal neuronal regions PY1-PY3, divided into equi-length areas, and CA4. Sections with Cu concentration and CCO activity are presented with demarcated ROI that correspond to regions of hippocampal anatomy. Pseudo-colored scale of Cu concentrations (μg/g) and gray scale (0-255) of relative optical density as measurement of CCO activity are presented between controls and epilepsy patients. # - an area with damaged tissue probably from cutting; data from this particular region were not included in further analysis. Scale bars: 1 mm. CV, cresyl violet; ROD, relative optical density; SUB, subiculum; PY1, PY2, PY3 - stratum pyramidale of cornuAmmonis (CA) fields 1, 2 and 3; CA4, CA field 4; RL, strata radiatum and lacunosum of CA; SL, stratum lucidum; M, stratum moleculare of CA; GDM, stratum moleculare of gyrusdentatus; GRCL, granular cell layer

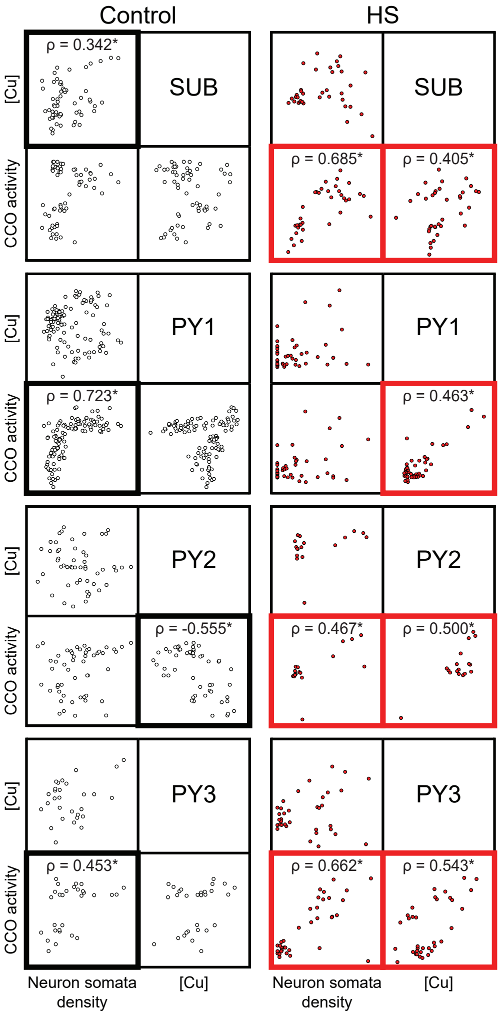

Control
HS
[Cu]
CCO activity
ρ = 0.342*
SUB
ρ = 0.685*
ρ = 0.405*
PY1
ρ = 0.723*
ρ = 0.463*
PY2
ρ = -0.555*
ρ = 0.467*
ρ = 0.500*
PY3
ρ = 0.453*
ρ = 0.662*
ρ = 0.543*
Neuron somata density
[Cu]

FIGURE S2 Histograms of neuron somata density per mm^2, Cu concentration and CCO activity in equi-length areas (500 µm each) along SUB and PY1-PY3 in hippocampi of control subjects (a), and mTLE-HS patients (b). # - an area with damaged tissue. SUB, subiculum; PY1, PY2, PY3 - stratum pyramidale of cornu Ammonis fields 1, 2 and 3

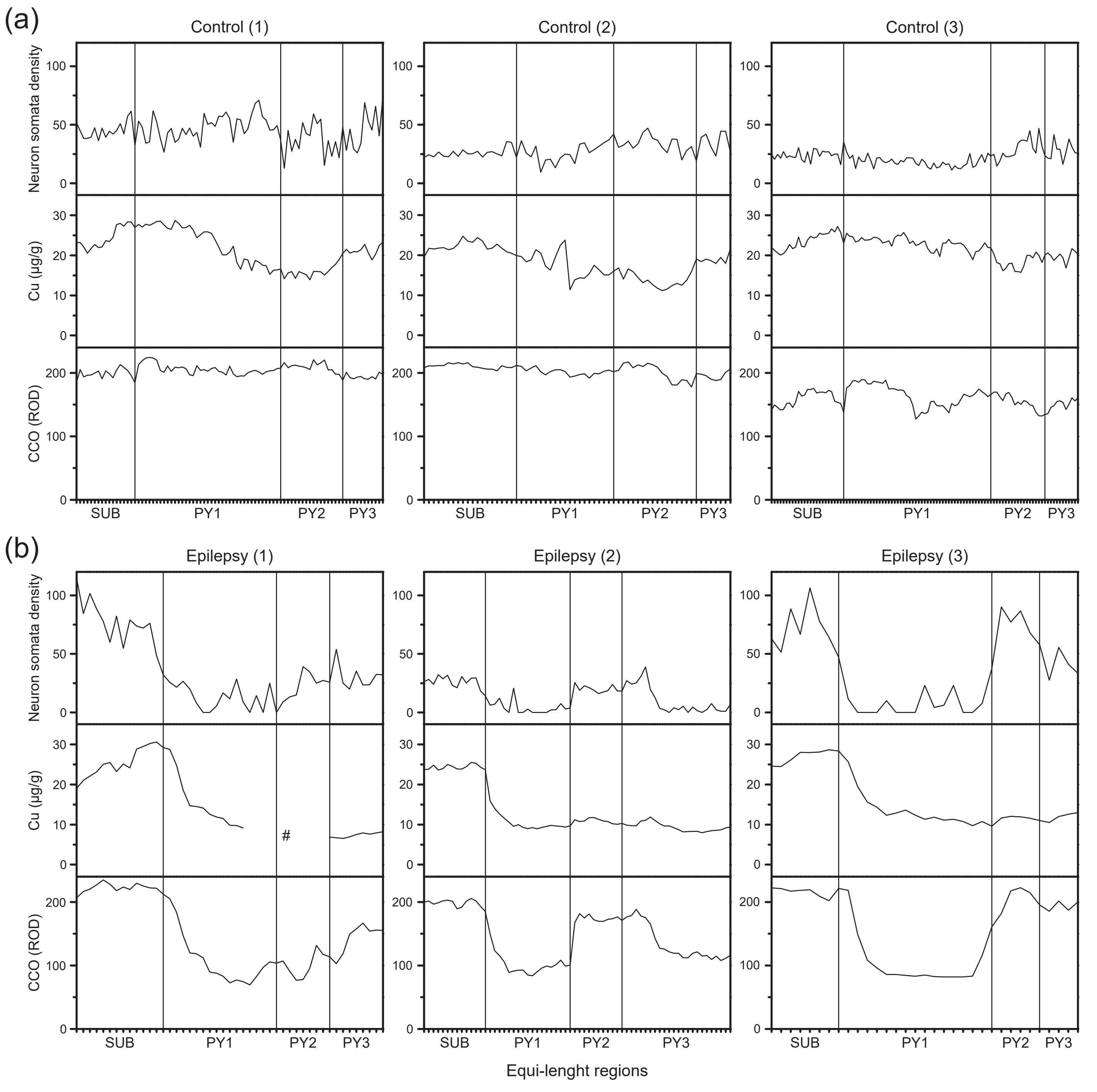

(a)
Control (1)
Control (2)
Control (3)
Neuron somata density
Cu (µg/g)
CCO (ROD)
SUB
PY1
PY2
PY3
(b)
Epilepsy (1)
Epilepsy (2)
Epilepsy (3)
#
Equi-lenght regions

FIGURE S3 Correlation analyses among neuron somata density (number of neurons/mm^2), Cu concentration [Cu] and CCO activity for SUB, PY1-PY3. Scatter plots of Spearman correlation analysis are presented in quadrants. ρ - Spearman's rank correlation coefficient. The relations were considered significant at $^*p<0.05$. SUB, subiculum; PY1, PY2, PY3 - stratum pyramidale of cornu Ammonis fields 1, 2 and 3

TABLE S1 Clinical data and characteristics of the analyzed subjects

Cryptonym	Sex	Age (years)	Epilepsy onset (age)	Epilepsy duration (years)	Febrile seizures	H of SE	sGTCS fr.	FCS fr.	AED trials	Seizure classification[a]	AH (side)	follow-up (months)	Outcome[b]	HS type[c]
E-1	Male	29	6	23	No	No	Never	Weekly	LEV, CBZ, PGB	Epigastric aura->automotor seizure	Right	54	III	1
E-2	Male	49	11	38	Yes	No	Monthly	Monthly	LEV, LTG, VPA, CBZ, PB, TPM	Automotor seizure->sGTCS	Left	14	III	1
E-3	Female	40	24	16	No	Yes	Monthly	Weekly	LEV, LTG, VPA, PGB, PHB, CBZ	Automotor seizure->sGTCS	Left	68	III	1
		Mean 39.3±5.8	Mean 13.7±5.4	Mean 25.7±6.5								Mean 45.3±16.2		
C-1	Male	19												
C-2	Male	19												
C-3	Female	38												
		Mean 25.3±6.3												

Abbreviations: E, epilepsy; C, control; H of SE, history of status epilepticus; sGTCS fr., secondary generalized tonic-clonic seizures frequency; FCS fr., focal complex seizures frequency; AED, antiepileptic drug; LEV, Levetiracetam; CBZ, Carbamazepine; PGB, Pregabaline; LTG, Lamotrigine; VPA, Valproic acid; PHB, Phenobarbital; TPM, Topiramate; AH, amygdalohippocampectomy; HS, hippocampal sclerosis.

[a] Lüders, H., Acharya, J., Baumgartner, C., Benbadis, S., Bleasel, A., Burgess, R., ... Wyllie, E. (1998). Semiological seizure classification. Epilepsia, 39, 1006–1013. https://doi.org/10.1111/j.1528-1157.1998.tb01452.x

[b] Wieser, H. G., Blume, W. T., Fish, D., Goldensohn, E., Hufnagel, A., King, D., ... Commission on Neurosurgery of the International League Against Epilepsy (ILAE) (2001). ILAE Commission Report. Proposal for a new classification of outcome with respect to epileptic seizures following epilepsy surgery. Epilepsia, 42, 282–286. https://doi.org/10.1046/j.1528-1157.2001.35100.x

[c] Blümcke, I., Thom, M., Aronica, E., Armstrong, D. D., Bartolomei, F., Bernasconi, A., ... Spreafico, R. (2013). International consensus classification of hippocampal sclerosis in temporal lobe epilepsy: A Task Force report from the ILAE Commission on Diagnostic Methods. Epilepsia, 54, 1315–1329. https://doi.org/10.1111/epi.12220

TABLE S2 The comparison of Cu concentrations [Cu] and relative CCO activity between SUB and other hippocampal regions in controls and patients (HS)

		SUB vs.							
	**Group**	**PY1**	**PY2**	**PY3**	**CA4**	**RL**	**M**	**GDM**	**GRCL**
**Neuron density**	Control	ns	ns	ns	ns				
	HS	*	ns	ns	*				
**[Cu]**	Control	ns	*	ns	ns	*	*	*	*
	HS	*	*	*	*	*	*	*	*
**CCO activity**	Control	ns	ns	ns	ns	ns	ns	ns	ns
	HS	*	ns	ns	*	*	*	ns	*

Note. * indicates statisticaly significant decrease (at $p<0.05$) as determined by one-way ANOVA followed by Duncan's post hoc test.

Abbreviations: HS, hippocampal sclerosis; CCO, cytochrome c oxidase; SUB, subiculum; PY1, PY2 and PY3, stratum pyramidale of cornu Ammonis (CA) fields 1, 2 and 3; CA4, CA field 4; RL, strata radiatum and lacunosum of CA; M, stratum moleculare of CA; GDM, stratum moleculare of gyrus dentatus; GRCL, granular cell layer.
